# Oncologic Outcomes in Patients with Localized, Primary Head and Neck Synovial Sarcoma

**DOI:** 10.3390/cancers16234119

**Published:** 2024-12-09

**Authors:** Riddhi R. Patel, Vancheswaran Gopalakrishnan, Behrang Amini, Alexander J. Lazar, Patrick P. Lin, Robert S. Benjamin, Andrew J. Bishop, Ryan P. Goepfert, Dejka M. Araujo

**Affiliations:** 1 Department of Sarcoma Medical Oncology, The University of Texas MD Anderson Cancer Center, |1515 Holcombe Blvd., Houston, TX 77030, USA; rpatel9@mdanderson.org (R.R.P.); rbenjami@mdanderson.org (R.S.B.); 2 Division of Epidemiology, The University of Texas School of Public Health, 1200 Pressler St., Houston, TX 77030, USA; 3 Division of Surgical Oncology, The University of Texas MD Anderson Cancer Center, 1515 Holcombe Blvd., Houston, TX 77030, USA; vancheswaran.gopalakrishnan@astrazeneca.com; 4 Division of Diagnostic Radiology-Musculoskeletal Imaging, The University of Texas MD Anderson Cancer Center, 1515 Holcombe Blvd., Houston, TX 77030, USA; bamini@mdanderson.org; 5 Department of Pathology, The University of Texas MD Anderson Cancer Center, 1515 Holcombe Blvd., Houston, TX 77030, USA; alazar@mdanderson.org; 6 Department of Orthopedic Oncology, The University of Texas MD Anderson Cancer Center, 1515 Holcombe Blvd., Houston, TX 77030, USA; plin@mdanderson.org; 7 Department of Radiation Oncology, The University of Texas MD Anderson Cancer Center, 1515 Holcombe Blvd., Houston, TX 77030, USA; 8 Department of Head and Neck Surgery, The University of Texas MD Anderson Cancer Center, 1515 Holcombe Blvd., Houston, TX 77030, USA; rgoepfert@mdanderson.org

**Keywords:** head and neck, synovial sarcoma, localized disease, chemotherapy

## Abstract

**Background:** this study aims to evaluate the survival outcomes of patients suffering from head and neck synovial sarcoma (HNSS), especially in relation to patients with a localized disease at diagnosis. **Methods:** this retrospective chart review includes 57 patients diagnosed with primary HNSS between 1981 and 2020 who presented with a localized disease at diagnosis. Overall survival (OS) from diagnosis, local recurrence-free survival (LRFS), and metastasis-free survival (MFS) from the end of the primary tumor treatment are estimated. The Kaplan–Meier method, the log-rank test, and the Cox proportional hazards regression are used. **Results:** the 5-year OS, LRFS, and MFS are estimated at 80.4% (95% CI: 66.6%, 88.9%), 67.7% (95% CI: 50.0%, 80.4%), and 50.6% (95% CI: 34.4%, 64.8), respectively. Compared to patients undergoing surgical resection alone, those receiving radiation therapy (RT) with surgery have better LRFS (HR: 0.03, 95% CI: 0.001, 0.57), and those undergoing neo/adjuvant chemotherapy with surgery and RT have better MFS (HR: 0.10, 95% CI: 0.01, 0.95). Moreover, among the patients with tumors ≥ 4 cm, those subject to neo/adjuvant chemotherapy have significantly better MFS (5-year MFS: 53.2%, 95% CI: 29.0%, 72.5%) than those treated with surgery and RT alone (5-year MFS: 20.0%, 95% CI: 0.8%, 58.2%) (LR-*p* = 0.003). **Conclusions:** overall, the prognosis of HNSS patients looks favorable. Perioperative RT significantly improves local control, and perioperative chemotherapy plays a vital role in delaying metastasis formation in patients with primary HNSS when diagnosed with a localized disease. Importantly, we recommend that systemic therapy should be considered for HNSS patients with tumors ≥ 4 cm.

## Simple Summary

Synovial sarcoma in the head and neck region is rare, making up just 2–3% of sarcomas in that area. This anatomic location is challenging to treat due to its proximity to vital structures like the airways and cranial nerves. Studying this rare malignant tumor at an early, localized stage is important, as multidisciplinary treatments might improve survival outcomes. Therefore, this study aims to explore the survival outcomes of patients with localized head and neck synovial sarcomas. We find that radiation therapy is associated with improved local control of the tumor. While the role of perioperative chemotherapy is still debatable in synovial sarcoma patients, we observe that undergoing chemotherapy in addition to surgery and radiation can promote metastasis-free survival in patients with localized head and neck synovial sarcomas, especially when the tumor is ≥4 cm. By providing updated information on this rare cancer type, such findings may help improve treatment approaches and outcomes, benefiting both patients and the medical community.

## 1. Introduction

Synovial sarcoma (SS) is a subtype of the soft-tissue sarcoma, commonly affecting young and middle-aged adults [1,2]. Despite its name, which reflects its microscopic resemblance to synovial tissue, SS originates from pluripotent mesenchymal cells rather than synovial structures [3,4]. SS represents about 8% of all soft-tissue sarcomas, with a predilection for the extremities [3]. Although histological grading criteria based on mitotic activity and tumor necrosis exist, SS is generally regarded as a high-grade tumor [5,6]. The tumor can be classified into monophasic or biphasic subtypes, depending on whether it contains solely spindle cells or both epithelial and spindle cells, with the monophasic form being more prevalent. Approximately 90% of SS cases exhibit the characteristic chromosomal translocation t(X;18), leading to a fusion between the *SYT1* gene on chromosome 18 and the *SSX1* or *SSX2* gene on chromosome X [2,6,7]. SS frequently metastasizes to the lungs, and 10-year survival rates are typically below 50% [8,9,10]. The recommended treatment approach includes wide local excision, often combined with radiation therapy (RT) and chemotherapy [1].

SS is uncommon in the head and neck region, accounting for only 2–3% of sarcomas in this area [2,11]. The 5-year limited duration prevalence rate of head and neck synovial sarcomas (HNSS) has been estimated at 0.04 per 100,000 people, with 34 estimated cases on 1 January 2020 in the US [12]. Compared to SS in other locations, HNSS is thought to be associated with a higher risk of regional and distant metastasis, primarily spreading through hematogenous routes [13,14]. HNSS tends to occur more frequently in men and typically presents in middle-aged individuals [15]. The soft tissues of the upper aerodigestive tract and neck are the most common primary sites for HNSS [2,7,15,16,17]. Clinically, HNSS often presents as a painless mass with or without symptoms such as hoarseness, dysphagia, odynophagia, or bleeding [17,18,19,20,21]. HNSS often presents unique diagnostic and treatment challenges due to its proximity to vital structures like the airways, nerves, and blood vessels. As with other SS cases, treatment for HNSS generally involves wide local excision alongside neo/adjuvant RT and chemotherapy [15].

Prognostic data for HNSS remain limited, particularly in cases presenting with a localized disease at diagnosis. Studying this rare malignant tumor at an early localized stage is important, as multidisciplinary treatments might improve survival outcomes. It is commonly assumed that HNSS carries a worse prognosis compared to SS at other sites. However, some reports suggest that HNSS may have a better prognosis than SS in the extremities, with overall survival rates ranging from 47% to 82% [16]. Given the scarcity of information and the conflicting data on localized HNSS prognoses, we aim to provide an updated analysis of the clinical characteristics and survival outcomes in patients with this rare malignancy.

## 2. Methods

This retrospective chart review was approved by the Institutional Review Board at The University of Texas MD Anderson Cancer Center (MD Anderson) in Houston, TX, USA. A total of 57 patients diagnosed with primary SS in the head and neck region between 1981 and 2020 who had a localized disease at the time of diagnosis and who presented to this tertiary cancer center were included. HNSS patients with a regional or distant metastatic disease at diagnosis were excluded from the study. Clinical and demographic information, including age at diagnosis (<15, 15–39, ≥40 years) [22], sex (female, male), race/ethnicity (white, other), tumor size (<5, 5–10, ≥10 cm), histological subtype (monophasic, biphasic, poorly differentiated), treatment (surgery only, surgery + RT, surgery + chemotherapy, surgery + RT + chemotherapy, RT + chemotherapy, and chemotherapy), and final surgical margins (positive, negative), was obtained from the institutional medical records and database.

### Statistical Analysis

There were three time-to-event outcomes: (1) overall survival (OS), defined as the time interval between diagnosis and death from all causes, (2) local recurrence-free survival (LRFS), defined as the time interval between the completion of the primary tumor treatment (surgery, radiation, or chemotherapy, whichever occurred last) and the first local recurrence, and (3) metastasis-free survival (MFS), defined as the time interval between the completion of the primary tumor treatment (surgery, radiation, or chemotherapy, whichever occurred last) and the first metastasis. Patients who were lost to follow-up were censored at their last contact date. All the outcomes were estimated using the Kaplan–Meier method. The effect of various factors on survival was determined using the log-rank test. Hazard ratios (HR) and 95% confidence intervals (CI) were estimated using the Cox proportional hazards regression. The multivariate models included significant variables from the univariate analysis. For LRFS and MFS, tumor size was included in the final model based on the log-rank *p* value. The analyses were conducted using STATA 17 (StataCorp, College Station, TX, USA). A *p* value lower than 0.05 was considered to be statistically significant.

## 3. Results

The median age at diagnosis in this cohort was 28 years, and most patients were men (68%) and white (70%). The majority of patients presented with a ≥ 5 cm tumor size (56%). Most of the primary tumors were located in the soft tissues of the neck (49%), followed by pharynx/larynx (23%), face (21%), and oral cavity (7%). About 49% of the patients had a monophasic subtype. Out of the 57 patients, 37 (65%) were subject to RT and chemotherapy with surgical resection, and 13 (23%) were subject to RT with surgery without chemotherapy (Table 1). Also, 11 out of 12 (92%) patients with positive margins underwent RT. The most common perioperative chemotherapy was doxorubicin and ifosfamide (83%, *n* = 33/40), with a median of five cycles.

**Figure 1:**
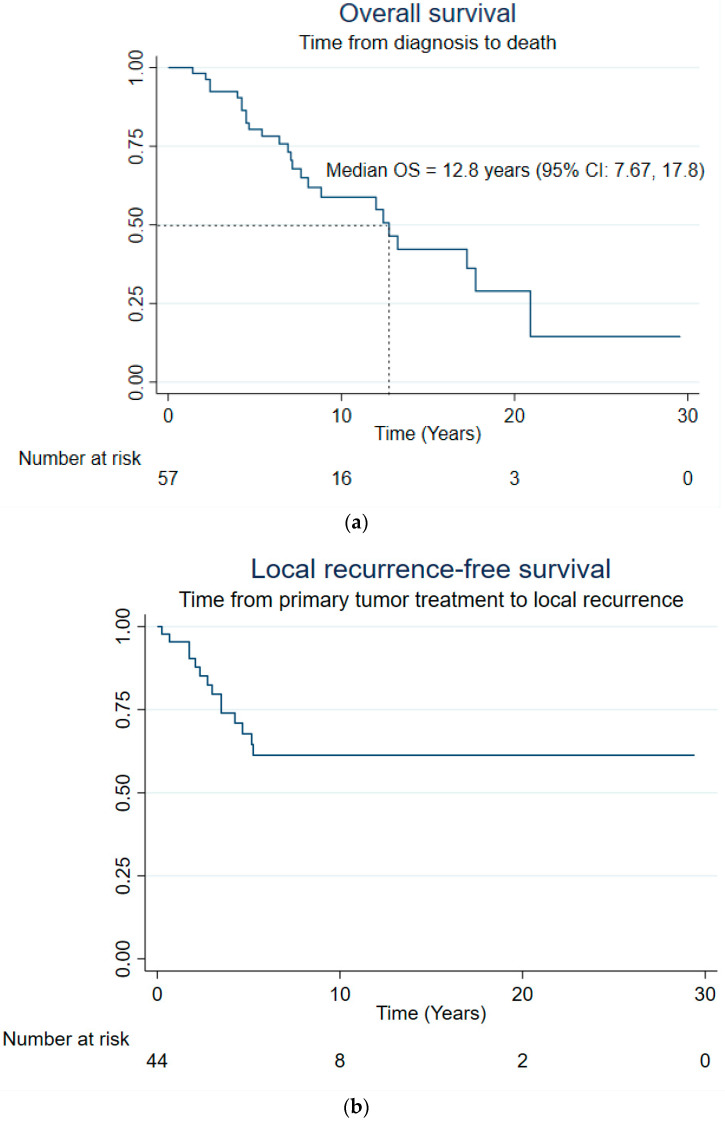

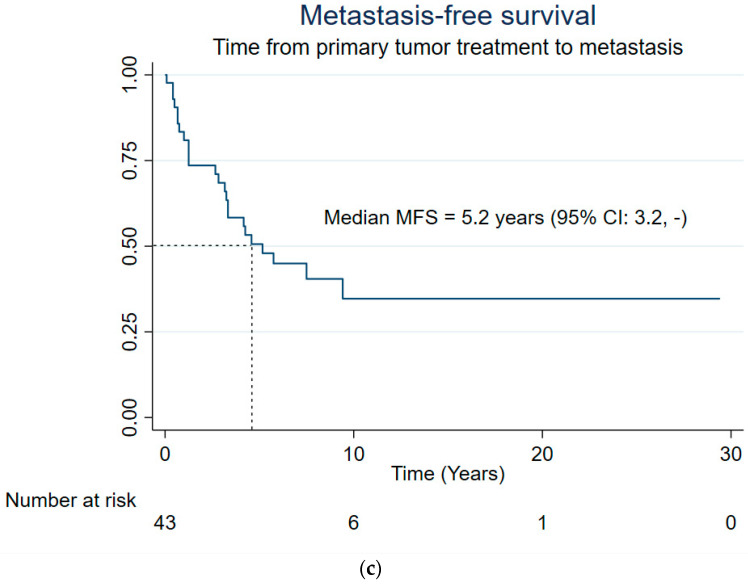
Overall survival (**a**), local recurrence-free survival (**b**), metastasis-free survival (**c**) curves.

A total of 25 patients out of 57 (44%) died. The median OS was 12.8 years (95% CI: 7.7, 17.8) (Figure 1a). The OS rates after 2 years, 5 years, and 10 years were 98.2% (95% CI: 87.6%, 99.7%), 80.4% (95% CI: 66.6%, 88.9%), and 58.8% (95% CI: 42.1%, 72.2%), respectively.

Of the 57 patients, 17 (30%) developed local recurrence and 30 (53%) developed metastases. The median LRFS was not reached, whereas the median MFS was estimated at 5.2 years (95% CI: 3.2, -) (Figure 1b,c). The LRFS rates after 2 years, 5 years, and 10 years were 90.4% (95% CI: 76.3%, 96.3%), 67.7% (95% CI: 50.0%, 80.4%), and 61.3% (95% CI: 43.2%, 75.2%), respectively. The MFS rates after 2 years, 5 years, and 10 years were 73.6% (95% CI: 57.4%, 84.4%), 50.6% (95% CI: 34.4%, 64.8%), and 34.7% (95% CI: 18.2%, 51.8%), respectively.

The median OS of diagnosed patients aged 15–39 years was significantly better (13.3 years) than that of the patients diagnosed at or after 40 years of age (6.9 years, log-rank *p* = 0.009) (Table 2). Based on the univariate Cox proportional hazards regression, being older than 40 years of age at diagnosis (HR: 10.66, 95% CI: 1.23, 92.16), younger than 15 years of age at diagnosis, and being white (HR: 5.66, 95% CI: 1.33, 24.09) rather than belonging to other races/ethnicities were associated with an increased risk of death (Table 3). However, both age and race were found to be statistically non-significant through multivariate analysis.

The treatment was found to be associated with both LRFS and MFS through multivariate analysis. Compared to patients who had undergone surgical resection alone, those who had been subject to RT with surgery experienced a 0.03-fold reduction in the risk of developing local recurrence (95% CI: 0.001, 0.57), while those who had undergone chemotherapy in addition to surgery and RT experienced a 0.02-fold reduction in the same risk (95% CI: 0.001, 0.34), after adjusting for tumor size (Table 3). Moreover, patients who had received neo/adjuvant systemic therapy with surgery and RT experienced a 0.10-fold reduction in the risk of developing metastases (95% CI: 0.01, 0.95) compared to those who had received surgical resection alone, after adjusting for tumor size (Table 3). The treatment-related results should be interpreted with caution, as the surgery only reference group contained only two patients (Table 1). Additionally, tumor size was also a predictor of MFS (HR for ≥5 cm size: 4.50, 95% CI: 1.14, 17.79) (Table 3).

## 4. Discussion

Even though SS is rare in the head and neck region, the current study is able to present detailed information on 57 patients with primary HNSS who presented with a localized disease at the time of diagnosis. This study’s findings suggest that HNSS has a relatively favorable prognosis compared to other primary SS locations.

In general, HNSS patients have better survival outcomes. As shown in Table 4, Wushou et al. found a 5-year OS of 81.4% among surgically treated patients, an OS estimate close to the 80.4% found in the current study [15]. As most of the patients in the current study had undergone surgical resection, the results of the concordant rates are comparable. Moreover, all the 5-year OS rates presented by Harb et al. (72%) [2], Mallen St. Clair et al. (66%) [16], and Aytekin et al. (70.5%) [23] fall in the 95% confidence interval (66.6%, 88.9%) found in the current study.

The median age at diagnosis found in this study’s cohort (i.e., 28 years) and the male predominance are consistent with the literature. Through univariate analysis, age at diagnosis and race were found to be significant predictors of OS among HNSS patients. Sultan et al., who studied SS among children and adults, found significantly higher mortality rates in adults than in children [24]. Older patients might experience a delay in diagnosis due to the rarity of the disease and the overlap of symptoms with more common head and neck conditions. This delay can lead to the disease being diagnosed at a more advanced stage, possibly affecting negatively the prognosis. Moreover, this study included a relatively higher proportion of white patients. This can be justified by the fact that white individuals have a higher likelihood of being diagnosed with SS compared to black individuals [2]. Interestingly, in this study, white patients had a worse prognosis than patients from other ethnic backgrounds. Although one might assume that white individuals, as a group, enjoy better access to healthcare, the underlying reasons for this finding remain unclear.

HNSS can affect different sites within the head and neck region. The most common site of tumor origin in this location is the parapharyngeal space in the neck [11,25,26]. In this region, the upper aerodigestive tract is also a frequent origin site for this tumor presentation [2,20]. A similar finding was observed in the current study, with 49% of the tumors arising within the neck. Notably, this study found that the OS, LRFS, and MFS did not differ across sites within the head and neck region. Therefore, tumors arising anywhere within the head and neck region should be treated as high risk.

Our usual treatment algorithm for HNSS is surgery with RT and sequencing based on discussions from our multidisciplinary team. In a study conducted by Mallen St. Clair et al. which included 167 HNSS patients, about 90% of the patients had surgery, and 65% of the patients had RT [16]. Negative surgical resection margins are generally correlated with a favorable prognosis in soft-tissue sarcoma patients [27,28]. Furthermore, negative resection margins among SS patients have been found to be highly associated with improved progression-free survival [29]. Although not significant in our study, we found that the median LRFS and MFS were better in patients with negative margins compared to patients with positive or uncertain margins. It is possible that RT may have potentially reduced the impact of positive margins in these patients.

Radiation therapy is often employed postoperatively to reduce the risk of local recurrence, especially when achieving clear surgical margins is challenging due to the anatomic complexity of the head and neck region. In this study, 80% of the patients were subject to adjuvant RT (Table 1). Moreover, both the patients who underwent RT and the patients who underwent chemotherapy with surgery and RT experienced a beneficial effect in relation to local recurrence. One of the reasons why chemotherapy can improve LRFS is that neoadjuvant chemotherapy can potentially shrink a tumor enough to make resection with negative margins more feasible. This can be inferred from the fact that 52% of the patients in this study had received neoadjuvant chemotherapy (Table 1).

The role of perioperative chemotherapy in SS, both site-specific and general, has long been the subject of debate in the literature. A meta-analysis of localized soft-tissue sarcomas has reported that adjuvant doxorubicin-based systemic therapy significantly contributes to improving time until local and distant recurrence in adult patients [30]. However, there is a lack of studies in the literature evaluating the role of neo/adjuvant chemotherapy in head and neck site-specific and localized disease-specific SS tumors because of insufficient data [31]. Notably, this study found that additional chemotherapy to the local resection with RT has a beneficial effect on LRFS and MFS.

**Figure 2:**
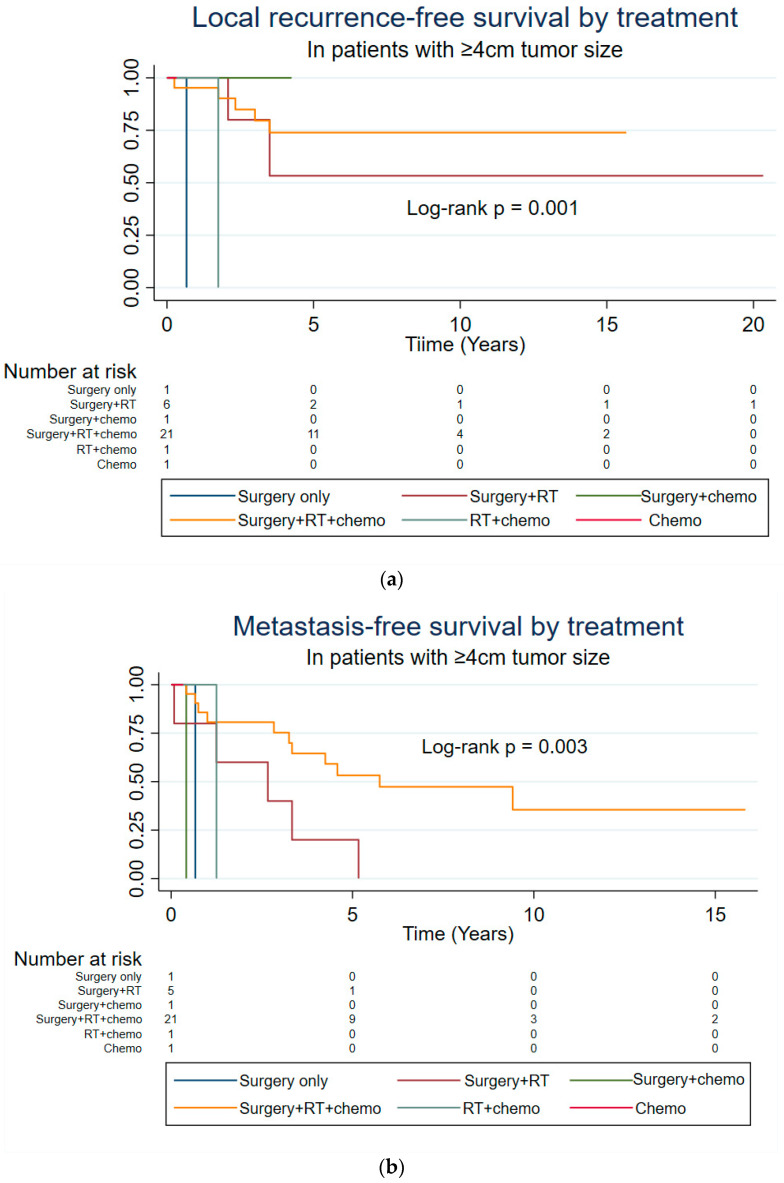
Local recurrence-free survival (**a**) and metastasis-free survival (**b**) according to treatment in patients with a tumor size ≥ 4 cm.

Upon further analysis, the authors observed that 16 out of the 57 patients who had undergone neo/adjuvant therapy in addition to surgery for the primary tumor had a tumor < 5 cm in size. Out of those 16 patients, 11 (69%) had undergone surgery + RT + chemotherapy (Table S1). This observation prompted the authors to explore the tumor size with a 4 cm cutoff. Following the performance of log-rank tests for treatment combinations in relation to tumor sizes < 4 and ≥4 cm, the results show that both LRFS and MFS were significantly different in patients with tumors ≥ 4 cm in size based on treatment combinations (Figure 2a: LRFS log-rank *p* = 0.001, and Figure 2b: MFS log-rank *p* = 0.003). Moreover, the 5-year LRFS of patients with a tumor size ≥ 4 cm who had undergone RT and surgery was 53.3% (95% CI: 6.8%, 86.3%), while the 5-year LRFS of patients who had undergone additional chemotherapy with surgery and RT was 74.9% (95% CI: 48.1%, 88.3%). Also, the 5-year MFS of patients with tumors ≥ 4 cm in size who had undergone RT with surgery was 20.0% (95% CI: 0.8%, 58.2%), while the 5-year MFS of patients who had been subject to additional chemotherapy with surgery and RT was 53.2% (95% CI: 29.0%, 72.5%). Based on these findings, we recommend considering neo/adjuvant systemic therapy for patients presenting with localized HNSS when the tumor size is 4 cm or larger. Additionally, the authors explored the characteristics of the patients experiencing a median OS of ≥12.8 years (*n* = 11) to potentially provide new insights for improving prognosis (Table S2). This analysis shows that four (36%) patients who survived for ≥12.8 years had been subject to RT and surgery, whereas seven (64%) patients had undergone chemotherapy in addition to surgery and RT (Table S2). In an era where the role of chemotherapy remains a topic of debate, this study underscores the critical importance of administering neo/adjuvant chemotherapy to HNSS patients.

There are several potential limitations pertaining to this study, including the retrospective data collection and the small sample size. Nevertheless, with regard to a rare disease, this is the largest cohort of HNSS patients from a single tertiary cancer center who presented with a localized disease at the time of diagnosis and provides comprehensive information on the survival outcomes. Moreover, approximately 40% of the cases in this cohort lacked data on fusion genes, representing a significant limitation in terms of both diagnostic and prognostic evaluations. However, the histological confirmation of all the cases was conducted by the dedicated soft-tissue sarcoma pathologist at our institution.

## 5. Conclusions

The overall prognosis of HNSS tumors appears to be good. The comparatively high 5- and 10-year OS, LRFS, and MFS rates in patients with HNSS indicate a fair-to-good prognosis for these tumors, despite the substantial treatment challenges posed by the occurrence of these tumors in the head and neck region. Perioperative RT improves local control. Remarkably, perioperative chemotherapy plays a significant role in delaying metastasis formation from definitive local therapy for the primary HNSS tumors. Therefore, we recommend that all HNSS patients receive treatment at a specialized sarcoma center with a multidisciplinary team of experts and that systemic therapy be considered for HNSS patients with tumors of 4 cm or larger.

## Figures and Tables

**Table 1:** Demographic and clinical characteristics of head and neck synovial sarcoma patients presenting with a localized disease at diagnosis (*n* = 57).

Characteristics	*n* (%)
Age at diagnosis, y median (range)	28 (5–66)
<15	5 (9)
15–39	41 (73)
≥40	11 (19)
Sex	
Female	18 (32)
Male	39 (68)
Race and ethnicity	
White	40 (70)
Other	17 (30)
Tumor size, cm median (range)	5.4 (0.7–11)
<5	16 (28)
5–10	31 (54)
≥10	1 (2)
Unknown	9 (16)
Tumor size, cm	
<4	11 (19)
≥4	37 (65)
Unknown	9 (16)
Subtype	
Monophasic	28 (49)
Biphasic	16 (28)
Poorly differentiated	7 (12)
Unknown	6 (11)
Fusion gene	
Present	35 (61.4)
Not tested	22 (38.6)
Site within head and neck	
Face	12 (21)
Pharynx/larynx	13 (23)
Neck	28 (49)
Oral cavity	4 (7)
Final surgical resection margin	
Negative	27 (40)
Positive	12 (22)
Unknown	16 (30)
Total (who had surgery)	55
Treatment	
Surgery only	2 (4)
Surgery + RT	13 (23)
Surgery + chemotherapy	3 (5)
Surgery + RT + chemotherapy	37 (65)
RT + chemotherapy	1 (2)
Chemotherapy	1 (2)
RT median dose (Gy) Timing of RT	59.9 (45–72)
Neoadjuvant	10 (20)
Adjuvant	40 (80)
Timing of chemotherapy	
Neoadjuvant	21(52)
Adjuvant	19 (48)

**Table 2:** Comparison of median survival times of head and neck synovial sarcoma patients presenting with a localized disease at diagnosis.

Characteristics	Median OS Log-Rank *p* Value Years	Median LRFS Log-Rank *p* Value Years	Median MFS Log-Rank *p* Value Years
Age at diagnosis, y	**0.009**	0.462	0.274
<15	NA	4.3	5.8
15–39	13.3	NA	7.5
≥40	6.9	NA	3.2
Sex	0.373	0.824	0.806
Female	12.8	NA	4.6
Male	8.8	NA	5.2
Race and ethnicity	**0.008**	0.427	0.1000
White	12.0	NA	5.8
Other	NA	NA	5.2
Tumor size, cm	0.433	0.500	**0.048**
<5	8.1	NA	NA
≥5	13.3	NA	3.3
Tumor size, cm	0.406	0.807	0.111
<4	7.2	5.3	NA
≥4	13.3	NA	3.3
Subtype	0.375	0.386	0.291
Monophasic	13.3	NA	5.8
Biphasic	7.2	NA	5.2
Poorly differentiated	17.8	NA	NA
Fusion gene	0.831	0.968	0.667
Present	12.4	NA	5.2
Not tested	12.8	NA	5.8
Site within head and neck	0.952	0.509	0.278
Face	8.8	NA	3.3
Pharynx/larynx	12.3	NA	NA
Neck	12.4	5.3	4.2
Oral cavity	17.3	1.8	1.3
Final surgical resection margin	0.300	0.121	0.172
Negative	NA	NA	NA
Positive/unknown	12.7	5.3	4.3
Treatment	0.202	**<0.001**	0.107
Surgery only	NA	NA	NA
Surgery + RT	17.3	NA	3.3
Surgery + chemotherapy	2.4	4.7	1.3
Surgery + RT + chemotherapy	8.8	NA	7.5
RT + chemotherapy	NA	NA	NA
Chemotherapy	NA	NA	NA
Bold values represent statistical significance with *p* < 0.05			

**Table 3:** Cox proportional hazards regression results for head and neck synovial sarcoma patients.

Characteristics	OS		LRFS		MFS	
	Unadjusted HR (95% CI)	Adjusted HR (95% CI)	Unadjusted HR (95% CI)	Adjusted HR (95% CI)	Unadjusted HR (95% CI)	Adjusted HR (95% CI)
Age at diagnosis, y						
<15	Reference	Reference	Reference	-	Reference	-
15–39	3.68 (0.47, 29.00)	0.89 (0.05, 14.87)	0.57 (0.16, 2.10)	-	0.88 (0.25, 3.03)	-
≥40	**10.66 (1.23, 92.16)**	2.03 (0.10, 39.97)	-	-	1.65 (0.39, 7.04)	-
Sex						
Female	Reference	-	Reference	-	Reference	-
Male	1.49 (0.62, 3.58)	-	0.88 (0.29, 2.65)	-	0.90 (0.38, 2.11)	-
Race and ethnicity						
White	**5.66 (1.33, 24.09)**	4.88 (0.64, 37.20)	1.59 (0.50, 5.08)	-	1.00 (0.43, 2.34)	-
Other	Reference	Reference	Reference	-	Reference	-
Tumor size, cm						
<5	Reference	-	Reference	Reference	Reference	Reference
≥5	0.68 (0.26, 1.78)	-	1.56 (0.42, 5.79)	1.44 (0.36, 5.74)	3.21 (0.94, 10.96)	**4.50 (1.14, 17.79)**
Tumor size, cm						
<4	Reference	-	Reference		Reference	
≥4	0.65 (0.23, 1.83)	-	0.93 (0.25, 3.46)		3.08 (0.71, 13.33)	
Subtype						
Monophasic	Reference	-	Reference	-	Reference	-
Biphasic	1.72 (0.66, 4.47)	-	0.94 (0.33, 2.72)	-	0.91 (0.37, 2.24)	-
Poorly differentiated	0.70 (0.15, 3.31)	-	-	-	-	-
Site within head and neck						
Face	Reference	-	Reference	-	Reference	-
Pharynx/larynx	0.70 (0.20, 2.46)	-	0.70 (0.10, 5.00)	-	0.32 (0.07, 1.34)	-
Neck	0.77 (0.25, 2.32)	-	1.89 (0.41, 8.79)	-	0.96 (0.35, 2.67)	-
Oral cavity	0.77 (0.14, 4.19)	-	2.33 (0.21, 25.81)	-	0.55 (0.06, 4.81)	-
Final surgical resection margin						
Negative	Reference	-	Reference	-	Reference	-
Positive/unknown	1.54 (0.67, 3.54)	-	2.46 (0.76, 8.02)	-	1.80 (0.76, 4.25)	-
Treatment						
Surgery only	Reference	-	Reference	Reference	Reference	Reference
Surgery + RT	0.29 (0.03, 2.51)	-	**0.01 (0.001, 0.31)**	**0.03 (0.001, 0.57)**	0.16 (0.02, 1.51)	0.27 (0.03, 2.72)
Surgery + chemotheraphy	1.57 (0.10, 25.28)	-	**0.03 (0.001, 0.85)**	0.04 (0.001, 1.36)	0.24 (0.02, 2.94)	0.46 (0.02, 9.47)
Surgery + RT + chemotherapy	0.55 (0.07, 4.56)	-	**0.02 (0.001, 0.31)**	**0.02 (0.001, 0.34)**	**0.09 (0.01, 0.84)**	**0.10 (0.01, 0.95)**
RT + chemotherapy	2.36 (0.15, 37.78)	-	0.32 (0.01, 7.99)	0.32 (0.01, 8.00)	0.47 (0.03, 8.13)	0.45 (0.03, 8.05)
Chemotherapy	-	-	-	-	-	-

HR: hazard ratio; CI: confidence interval; Bold values represent statistical significance with *p*<0.05.

**Table 4:** Comparison of 5-year overall survival rates across HNSS studies.

Study	Cohort Size	Major Findings
Harb et al. [2] (2007)	N = 40	5-year OS = 72%
Wushou et al. [15] ^a^ (2015)	N = 93	Treated with surgery
		5-year OS = 81.4%
Mallen St. Clair et al. [16] ^b^ (2016)	N = 167	5-year OS = 66%
Aytekin et al. [23] ^c^ (2020)	N = 224	5-year OS = 70.5%
Current study	N = 57	Localized disease
		5-year OS = 80.4%

^a^ Meta-analysis. ^b,c^ SEER analysis.

## Data Availability

The data presented in this study are available upon request from the corresponding author due to privacy, legal, and ethical reasons.

## Supplementary Materials

The following supporting information can be downloaded at: https://www.mdpi.com/article/10.3390/cancers16234119/s1, Table S1. Treatment distribution by tumor size. Table S2. Comparison of characteristics for HNSS patients by median overall survival time (n = 57).
