# Supplementary material for: Oncologic Outcomes in Patients with Localized, Primary Head and Neck Synovial Sarcoma
Source: Cancers (Basel). 2024 Dec 9;16(23):4119. doi: 10.3390/cancers16234119 (PMC11639992; doi:10.3390/cancers16234119)
Supplement: Supplementary file 1 [file cancers-16-04119-s001.zip › cancers-3257105-supplementary.pdf]

Supplementary Tables:

Table S1. Treatment distribution by tumor size.

Treatment	Tumor size <5 cm (n = 16)	Tumor size ⩾5 cm (n = 32)	p-value
Surgery only	0	2 (6)	0.974
Surgery + RT	4 (25)	7 (22)	
Surgery + chemo	1 (6)	1 (3)	
Surgery + RT + chemo	11 (69)	20 (63)	
RT + chemo	0	1 (3)	
chemo	0	1 (3)	

Tumor size missing (n = 9)

Table S2. Comparison of characteristics for HNSS patients by median overall survival time (n = 57).

Characteristics	Patients who survived <12.8 years (n = 46)	Patients who survived ⩾ 12.8 years (n = 11)	P-value
Age at diagnosis, y median (range)	28 (12-66)	23 (5-41)	0.329
<15	3 (7)	2 (18)	
15-39	33 (72)	8 (73)	
≥40	10 (22)	1 (9)	
Sex			1.000
Female	15 (33)	3 (27)	
Male	31 (67)	8 (73)	
Race and ethnicity			0.717
White	33 (72)	7 (64)	
Others	13 (28)	4 (36)	
Tumor size*, cm median (range)	5.2 (0.7-11.0)	6 (2-9.1)	0.240
<5	15 (38)	1 (13)	
≥5	25 (63)	7 (88)	
Tumor size*, cm			0.661
<4	10 (25)	1 (13)	
≥4	30 (75)	7 (88)	
Subtype			0.415
Monophasic	23 (50)	5 (45)	
Biphasic	14 (30)	2 (18)	
Poorly differentiated	4 (9)	3 (27)	
Unknown	5 (11)	1 (9)	
Site within head and neck			0.094
Face	12 (26)	0	
Pharynx/Larynx	11 (24)	2 (18)	
Neck	21 (47)	7 (64)	
Oral Cavity	2 (4)	2 (18)	
Final surgical resection margin			0.787
Negative	22 (48)	5 (45)	
Positive/Unknown	22 (48)	6 (55)	
	2 (4) No surgery	-	
Treatment			0.812
Surgery only	2 (4)	0	
Surgery + RT	9 (20)	4 (36)	
Surgery + chemo	3 (7)	0	
Surgery + RT + chemo	30 (65)	7 (64)	
RT + chemo	1 (2)	0	
chemo	1 (2)	0	

*Tumor size missing for total of 9 patients.
